# Understanding the Molecular and Cell Biological Mechanisms of Electrical Synapse Formation

**DOI:** 10.3389/fnana.2020.00012

**Published:** 2020-04-15

**Authors:** E. Anne Martin, Abagael M. Lasseigne, Adam C. Miller

**Affiliations:** Department of Biology, Institute of Neuroscience, University of Oregon, Eugene, OR, United States

**Keywords:** electrical synapse, synaptogenesis, cell biology, connexin, Cx36, cytoskeleton, junction, zebrafish

## Abstract

In this review article, we will describe the recent advances made towards understanding the molecular and cell biological mechanisms of electrical synapse formation. New evidence indicates that electrical synapses, which are gap junctions between neurons, can have complex molecular compositions including protein asymmetries across joined cells, diverse morphological arrangements, and overlooked similarities with other junctions, all of which indicate new potential roles in neurodevelopmental disease. Aquatic organisms, and in particular the vertebrate zebrafish, have proven to be excellent models for elucidating the molecular mechanisms of electrical synapse formation. Zebrafish will serve as our main exemplar throughout this review and will be compared with other model organisms. We highlight the known cell biological processes that build neuronal gap junctions and compare these with the assemblies of adherens junctions, tight junctions, non-neuronal gap junctions, and chemical synapses to explore the unknown frontiers remaining in our understanding of the critical and ubiquitous electrical synapse.

## Introduction

**Figure 1:**
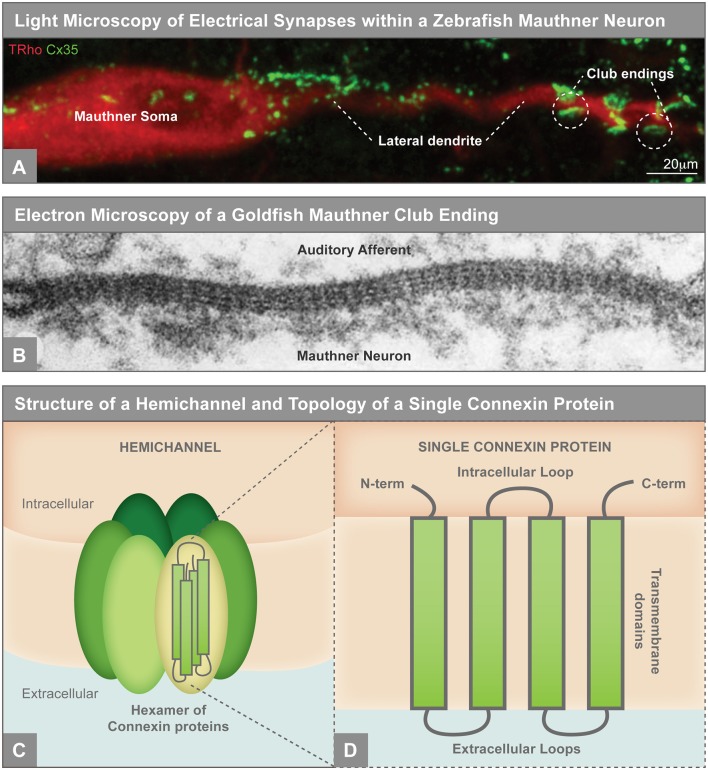
**(A)** Electrical synapses visualized by light microscopy on the larval zebrafish Mauthner neuron. Mauthner, labeled with tetramethylrhodamine-dextran (TRho, red), makes electrical synapses, labeled by Connexin35 (Cx35, green), on its soma and lateral dendrite. The so-called club ending synapses represent uniquely identifiable electrical connections with auditory afferents. The Mauthner neuron has served as a key model for electrical synapse formation and function and the principles learned have applied to both invertebrate and vertebrate systems (Nagy et al., 2018). Image modified from Yao et al. (2014), reproduced with permission. **(B)** Electron microscopy showing gap junctions at the club endings between the postsynaptic Mauthner neuron and the presynaptic auditory afferents in adult goldfish. The electron density between the neurons and the characteristic intermembrane spacing are hallmarks of gap junctions. X 285,000. Republished with permission of Rockefeller University Press, from Brightman and Reese (1969); permission conveyed through Copyright Clearance Center, Inc. **(C)** Illustration of an unpaired gap junction hemichannel inserted into the plasma membrane, composed of a hexamer of Connexin proteins. **(D)** A single Connexin protein is illustrated to show protein topology.

Electrical synapses are specialized connections between neurons that facilitate direct ionic and small metabolite communication (Figure 1). They are composed of tens to thousands of gap junction channels clustered together into plaques that are present throughout developing and adult brains. Electrical synapses contribute towards initial neural circuit function including driving the earliest animal behaviors (Rekling et al., 2000; Saint-Amant and Drapeau, 2000; Marin-Burgin et al., 2006; Su et al., 2017) and continue to function broadly throughout life in neural circuits controlling sensory processing (Li et al., 2009; Huang et al., 2010; Yaksi and Wilson, 2010; Pouille et al., 2017), rhythmic behavior in central pattern generators and motor systems (Eisen and Marder, 1982; Song et al., 2016; Traub et al., 2020), and cortical processing in mammals (Galarreta and Hestrin, 2001, 2002; Connors and Long, 2004; Gibson et al., 2005; Hestrin and Galarreta, 2005; Mancilla et al., 2007). Despite these well-documented and diverse circuit functions (reviewed in Nagy et al., 2018), the electrical synapse is commonly thought of as a necessary, but simple and temporary, precursor in development to the later-forming chemical synapse. However, emerging evidence supports an alternative view, namely that electrical and chemical synapses are essential life-long collaborators in both invertebrate and vertebrate neural circuits where they work synergistically to dynamically shape brain function (reviewed extensively in Pereda, 2014). Indeed, the best-studied electron-microscope reconstructed connectomes, of *C. elegans* and the rabbit retina, reveal that electrical synapses make up about 20% of connections in these mature circuits (White et al., 1986; Anderson et al., 2011; Jarrell et al., 2012; Cook et al., 2019). Also, electrical synapses have emerged as complex biochemical structures, with their proteomic diversity supporting sophisticated neuronal functions including activity-dependent plasticity (reviewed in Miller and Pereda, 2017). These findings lead to exciting new ideas about the role of electrical synapses in brain development, function, and disease. However, while abundant literature has explored the mechanisms that build both non-neuronal gap junction and chemical synapse formation, the field still has only furtive glances into the cell biological mechanisms that control electrical synapse formation and function. Given that electrical synapses are formed within the elaborate architecture of neurons and that they are optimized for fast transmission and plasticity, we expect that complex cell biological rules regulate the formation and homeostasis of these gap junction channels. Here we focus on emerging evidence that provides the first glimpse of electrical synapse cell biology *in vivo*. We apologize for the many excellent articles we were unable to cite in this review due to space constraints, but the explosion of renewed interest in these structures has generated many recent reviews that provide excellent resources to examine the many aspects of electrical synapse structure and function (Dong et al., 2018; Harris, 2018; Jabeen and Thirumalai, 2018; O’Brien and Bloomfield, 2018; Traub et al., 2018; Alcamí and Pereda, 2019; Totland et al., 2020).

### The Formation of Intercellular Junctions

**Figure 2:**
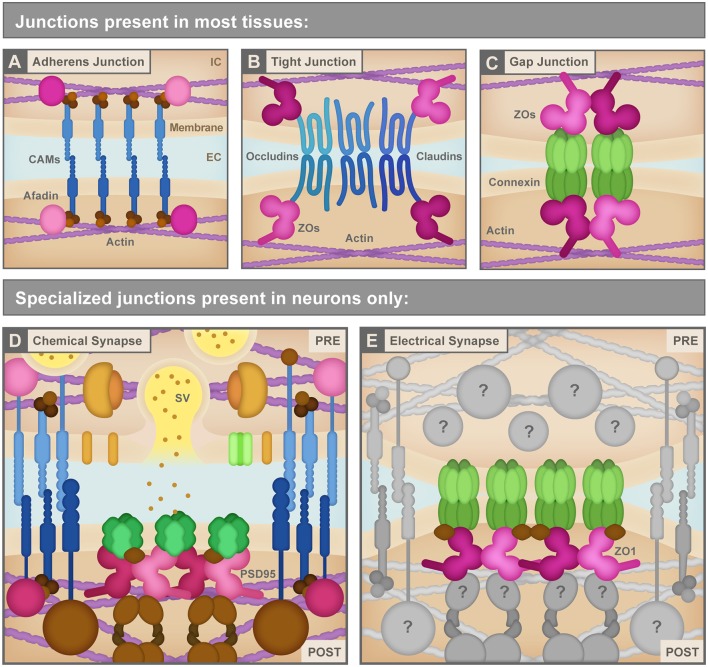
**(A)** Adherens junctions are the simplest junctions consisting of cell adhesion molecules (CAMs, blue) such as cadherins and nectins, and scaffolding proteins like Afadin (pink) combined with linker proteins (brown) such as catenins that connect cellular membranes to the actin cytoskeleton (purple). IC, Intracellular; EC, Extracellular. **(B)** Tight junctions use different CAMs (blue) including claudins and occludins to bring the neighboring cell membranes tightly together. These CAMs connect to the actin cytoskeleton (purple) *via* several scaffolding molecules (pink) including ZO proteins. **(C)** Non-neuronal gap junctions use Connexin proteins arranged in hexameric hemichannels (green) to intercellularly connect cells. Connexins also use scaffolding proteins (pink) including ZO proteins to link to other signaling molecules and the actin cytoskeleton (purple). **(D)** Chemical synapses, such as the glutamatergic excitatory chemical synapse represented here, have a vast assortment of proteins composing their structure including a variety of CAMs (blue), scaffolding molecules such as PSD95 (pink), neurotransmitters and synaptic vesicles (SV) and associated proteins (yellow and orange), neurotransmitter receptors and calcium channels (green), cytoskeletal adaptor proteins and other signaling molecules (brown), etc. PRE, Presynapse; POST, Postsynapse. **(E)** Electrical synapses are neuronal gap junction channels and use Connexins (green) to directly interconnect two neurons. Electrical synapses are often thought of as molecularly symmetric, but they can have asymmetric protein localization, as depicted here. At asymmetric electrical synapses, two postsynaptic proteins, ZO1 (pink) and Ca2+/calmodulin-dependent protein kinase II (CAMKII, brown) are observed to directly interact with Connexin C-terminal tails in the postsynapse to provide scaffolding and kinase activity. Due to the cell-biological specificity of electrical synapse formation within the complexity of neuronal morphology, and given their sophisticated functions in fast interneuronal communication, we expect that a large assortment of unknown proteins (gray) exists to manage electrical synapse formation and function. See the text for details. Republished with permission of Rockefeller University Press, from Brightman and Reese (1969); permission conveyed through Copyright Clearance Center, Inc.

While the mechanisms that build an electrical synapse are not well understood, critical clues to how the process might work are likely to be found in the known mechanisms that build other junction types such as adherens junctions, tight junctions, non-neuronal gap junctions, and chemical synapses. This process of junction formation requires: (1) selecting the junction site; (2) adhering to the cellular membranes in close apposition; (3) anchoring to the cytoskeleton; and (4) coordinating protein recruitment between the two cells to form a functional junction. Every junction type must create molecular solutions to these problems, and while each junction has its unique features, they share a common foundation (Figure 2).

In both neuronal and non-neuronal tissues, adherens, tight, and gap junctions exist to link cells to one another. Adherens junctions essentially take on the role of molecular glue between cells (Figure 2A). These structures mediate cell-cell adhesion *via* the extracellular binding of cell adhesion molecules (CAMs), which include transmembrane cadherins and nectins (Troyanovsky, 2014). Intracellularly, CAMs anchor the cell membrane to actin *via* cytoskeleton-interacting linkers and scaffolding proteins such as catenins and afadin (Indra et al., 2013). By contrast, tight junctions bind cells to one another to create a seal that generates a mesh-like barrier with small pores between tissues. These junctions largely use the claudin CAM family as their transcellular connector and link to intracellular scaffolding proteins such as ZO proteins (Figure 2B; Zihni et al., 2016). Unlike adherens and tight junctions, gap junctions create a physical intercellular channel connecting the two cell cytoplasms and making a direct passage for ions and other small molecules to pass from cell to cell. Gap junctions are created by coupled hemichannels contributed by each cell, with each hemichannel, in vertebrates, being comprised of a hexamer of Connexin proteins (Figures 1C, 2C). Invertebrates accomplish the same task by using an evolutionarily distinct class of proteins called Innexins to form gap junctions (reviewed in Phelan, 2005; Güiza et al., 2018). Much like the CAMs at adherens and tight junctions, Connexins are intracellularly connected to scaffolding and cytoskeletal linkage proteins including ZO proteins and EB1 (Li et al., 2004; Epifantseva and Shaw, 2018). Thus, while there is some molecular overlap, each junction’s unique morphology and function requires specialized membrane proteins, and fundamentally each must have a form of CAM, a scaffold, and an anchor to the cytoskeleton. How does this change within a neuronal environment?

Neurons use their special intercellular junctions to support the fast communication needs of neural network function. Moreover, the cell biological demands of their complex and diverse morphology (far-reaching axons and dendrites) require a carefully orchestrated protein delivery and control system (Tahirovic and Bradke, 2009). In particular, neuronal cells have two specialized junctions to manage fast information flow: chemical and electrical synapses. Chemical synapses (Figure 2D) are fundamentally asymmetric structures, with the presynaptic side, the so-called active zone, specialized for fast synaptic vesicle release in response to neuronal action potentials (Südhof, 2012). Synaptic vesicle exocytosis at the active zone releases neurotransmitters into the synaptic cleft between the neurons to activate receptors on the postsynaptic cell. The postsynapse also termed the postsynaptic density, is specialized to manage the localization, organization, and function of neurotransmitter receptors to control communication (Frank and Grant, 2017). As with their non-neuronal junction counterparts, common mechanistic themes control the formation of all chemical synapses. Synaptic CAMs are thought to initiate synaptogenesis and offer trans-synaptic structural support; intracellular synaptic scaffolding molecules organize and stabilize both the pre- and postsynaptic compartments; and adaptor proteins link to the cytoskeleton to manage trafficking, anchoring, and later plasticity. Proteomic work on pre- and postsynaptic chemical synapses have revealed hundreds and thousands of proteins, respectively, in each compartment (Collins et al., 2006; Bayés and Grant, 2009; Ryan and Grant, 2009; Dieterich and Kreutz, 2016). While there is great protein diversity in these connections, each of the molecular aspects of building a chemical synapse relates to the fundamental themes of adhesion, scaffolding, and cytoskeletal anchoring, and these are critical to the structure, function, and plasticity of these connections.

While we know relatively little about the molecular mechanisms that regulate electrical synapses (Figure 2E), their observed functional diversity and plasticity suggests complex cell biological rules must control their formation and function, presumably using similar mechanisms as the other junction types. The notion of electrical synapse complexity is supported by several observations. First, we know that these neuronal gap junctions appear throughout the nervous system, from sensory neurons to central processing circuits to motor outputs (Galarreta and Hestrin, 2001; Connors and Long, 2004; Nagy et al., 2018). Besides, circuits build these connections in development and then refine them to form the final set of electrical synapses used in adulthood (Rash et al., 2000; Galarreta and Hestrin, 2002; Pereda, 2014). Thus, there must exist critical gene regulatory networks controlling when and where electrical synapse genes are expressed. Second, electron microscopy shows that the cell biological construction of electrical synapses is varied, and these structures can form between all neuronal compartments: there are axo-dendritic, somato-somatic, axo-axonic, and dendro-dendritic electrical synapses (Kosaka and Hama, 1985; Hamzei-Sichani et al., 2007; Nagy et al., 2018). These varied configurations suggest molecular specificity mechanisms to ensure electrical synapses are made in the right places and at the right times. Finally, electrical synapses are found in multiple morphological arrangements, such as in dense plaques, lacey plaques, wide ribbons, and thin strings (Nagy et al., 2018), suggesting that individual synapses are differentially regulated to achieve their unique functional needs. Here, we will explore the cell biological and molecular mechanisms which likely exist to manage each of these processes, beginning with gene expression control, then how gap junction proteins arrive at the synapse, followed by an analysis of electrical synapse organization, then by addressing how an electrical synapse site may be specified, and finally by exploring how electrical synapses may contribute to disease. Our goal is to highlight critical areas of unexplored biology with the hope that this spurs efforts to identify the molecules and mechanisms that build, maintain, and allow for the modification of the electrical synapse.

## Expression and Localization of Gap Junction Forming Genes

To make electrical synapses, neurons must express genes that support gap junction formation. In chordates, gap junctions are created by Connexins while in non-chordate animals Innexins make the channels (Slivko-Koltchik et al., 2019). While chordates retain Innexin genes, called Pannexins in these genomes, these proteins only make hemichannels and do not form intercellular junctions (Abascal and Zardoya, 2013). Despite evolution devising two molecular solutions to forming gap junctions, Connexin and Innexin structure and function are strikingly conserved (Goodenough and Paul, 2009; Pereda and Macagno, 2017). All animal genomes contain large numbers of gap junction forming genes, each expressed in cell-type-specific patterns and encoding proteins that facilitate unique functions. Therefore, to understand the electrical synapses of the nervous system, it is critical to examine the molecular complexities of the gap junctions. In *C. elegans*, 17 of the 25 Innexin genes are neuronally expressed, and they display highly complex and overlapping patterns that suggest incredible electrical synapse molecular complexity (Bhattacharya et al., 2019). Analogously, vertebrate genomes encode many Connexins; for example, zebrafish contain ~40 unique genes (Watanabe, 2017). Most Connexin genes are not expressed within neurons, such as the gene *gap junction a1 (gja1)* encoding the Connexin43 (Cx43) protein, which is expressed in non-neuronal tissue including epithelia and glia (Janssen-Bienhold et al., 1998; Güldenagel et al., 2000; Misu et al., 2016). A subset of Connexins are expressed in neurons, though each gene has a unique expression profile within the nervous system (Li et al., 2009; Rash et al., 2013; Klaassen et al., 2016; Song et al., 2016; Miller et al., 2017). For example, the *gjd2*/Cx36 family of genes are the most broadly expressed neuronal Connexins, found in neurons from the forebrain to the spinal cord within zebrafish and mouse brains (Condorelli et al., 1998; Connors and Long, 2004; Li et al., 2009; Söhl et al., 2010; Miller et al., 2017). By contrast, the mammalian *gja10*/Cx57 gene and its homologs in zebrafish are expressed exclusively in retinal horizontal cells (Söhl et al., 2010; Klaassen et al., 2016; Greb et al., 2018). Thus, while a complete accounting of vertebrate Connexin expression in the nervous system has not yet been achieved, it is clear that regulated expression contributes to the specificity of the electrical connectome.

In addition to gene regulatory mechanisms contributing to electrical synapse specificity, there are complexities as to whether two different Connexins can form a gap junction. For example, Cx43 expressed within glia cannot form gap junctions with neuronally expressed Cx36 (Rash et al., 2001; Koval et al., 2014). By contrast, many Connexin types can interact with one another, either within a hemichannel or between apposed cells. Given that many neurons express multiple Connexin proteins, there is the potential for a variety of Connexin arrangements within neuronal gap junctions (O’Brien et al., 2004; Rash et al., 2013; Palacios-Prado et al., 2014; Miller et al., 2017). These rules of engagement are certainly important for creating specific connectivity, yet we still lack the complete set of compatibility guidelines between the large family of Connexins. The spatial and temporal control of Connexin expression, coupled with the rules of Connexin engagement, provide both specificity and opportunities for complexity in the formation of electrical synapses. Future work is required to elucidate the complete molecular map of electrical synapse gene expression and protein usage in a complex vertebrate brain such as zebrafish.

While Connexin incompatibilities and expression are important for specificity, it is also clear that neurons are selective in where they form electrical synapses. An intriguing example of this is found within the mouse retina where the rod and cone photoreceptors express Cx36 and make electrical synapses with one another (Deans et al., 2002; Li et al., 2014; Asteriti et al., 2017). The photoreceptors also make chemical synapses with bipolar neurons, which themselves are coupled to other retinal neurons by Cx36-mediated electrical synapses (Deans et al., 2002; Trenholm and Awatramani, 2019). However, the photoreceptors do not make electrical synapses with bipolar neurons, despite their ability to form chemical synapses with one another and their mutual expression of Cx36. How can this be? The answer must arise from cell biological mechanisms that specify where the Connexins travel within the cell to form gap junctions. Yet we know little about the trafficking mechanisms of Connexins within neurons.

## Trafficking of Connexins within Neuronal Compartments

Most of our understanding of Connexin trafficking comes from studies of Cx43-based gap junctions (reviewed in Epifantseva and Shaw, 2018). In essence, Cx43 hemichannels are packaged into vesicles, travel along microtubules to an adherens junction situated near an established gap junction plaque, and are deposited into the membrane where they then migrate to and are incorporated into the plaque. However, in considering how electrical synapses are built, neurons offer additional trafficking challenges given their distinct cellular compartments. In most vertebrate neurons, axons are far-reaching processes that control information transmission at the presynapse, while dendrites are highly branched processes that typically stay relatively near the cell soma and manage information reception at the postsynapse. Axons and dendrites use analogous yet distinct processes to manage specific protein trafficking to their pre- and postsynaptic contact points. While chemical synapse contacts are necessarily asymmetric, electrical synapses can be either symmetric or asymmetric, and the flow of information at the electrical synapse can be bi-directional or biased (rectified; Phelan et al., 2008; Rash et al., 2013; Miller et al., 2017; Bhattacharya et al., 2019). In this review article, we will often refer to presynaptic (axonal) and postsynaptic (dendritic) electrical synapse components, and we do so only concerning the polarized neuronal compartments in which each side of the synapse resides. Given that electrical synapses occur on dendrites, cell bodies, and axons, and that axons and dendrites use different methods to traffic proteins, the trafficking of Connexins and other electrical synapse components within neurons must be controlled to build the appropriate electrical connections.

A striking example of the molecular organization of Connexins within distinct neuronal compartments was recently revealed using the power of zebrafish genetics. In zebrafish Mauthner neurons, two Connexins, Cx34.1 and Cx35.5, both homologous to mammalian Cx36, are necessary for electrical synapse formation (Miller et al., 2017). Surprisingly, Cx34.1 is specifically required in the postsynapse while Cx35.5 is exclusively required in the presynapse, but the mechanisms guiding compartment-specific Connexin localization are unknown. This asymmetric compartmentalization of Connexins suggests that molecular rules must exist to guide specific Connexin types to particular sub-neuronal regions. Connexin proteins are four-pass transmembrane domain proteins with N- and C-termini located intracellularly (Figure 1D). Postsynaptic Cx34.1 and presynaptic Cx35.5 are ~90% amino acid identical, yet they have tantalizing differences in their intracellular loops and C-terminal tails which must, in some as yet undiscovered way, support their separate requirement in dendrites and axons. If we look to the chemical synapse for clues, we find that the trafficking and stabilization of postsynaptic AMPA neurotransmitter receptor subtypes are regulated through interactions between its C-terminal domain and intracellular scaffolding proteins, which connects them to the cytoskeleton and other signaling molecules (reviewed in Anggono and Huganir, 2012). But how do neurons target Connexins to these different neuronal compartments?

To traffic along axons and dendrites, Connexins first need to be packaged into vesicles which sort them into neuronal compartments according to the proteins on the vesicle surface. Identifying the types of vesicles in which Connexins transit would help us to understand their trafficking pathway, but these vesicles are yet to be identified. The vesicles must next engage with the intrinsic neuronal polarity mechanisms that define dendrites and axons, particularly the motor proteins that direct traffic along microtubules to these specific regions. These compartmental motors are distinctly organized: guidance to the presynapse along the axon requires kinesin motor proteins, and guidance to the postsynapse along the dendrite requires tethering to both kinesins and dyneins, with short-range, synaptic delivery in each compartment guided by actin-trafficked myosin motor proteins (for a detailed analysis of axon and dendrite polarity differences see Rolls and Jegla, 2015). Both tubulin (Brown et al., 2019) and actin (Wang, 2015) are required for proper trafficking of Cx36 to the membrane. Yet we still do not know the types of motor proteins Connexins or other electrical synapse components use to direct electrical synapse protein trafficking. However, recently some clues have started to point the field in the right direction.

Connexins likely rely on adaptor proteins to regulate their transport to the synapse. In a forward genetic screen using zebrafish, the epilepsy- and autism-associated gene Neurobeachin was identified as necessary for both electrical and chemical synapse formation (Iossifov et al., 2014; Miller et al., 2015; Mulhern et al., 2018). Neurobeachin is localized on vesicles which are found at the trans side of the Golgi, along dendrites, and also at chemical postsynapses (Wang et al., 2000; Miller et al., 2015). Its localization at electrical synapses is currently unknown. Past studies show Neurobeachin regulates membrane protein trafficking of chemical synapse scaffolds including PSD95 and SAP102 which in turn control the trafficking of neurotransmitter receptors (Medrihan et al., 2009; Niesmann et al., 2011; Nair et al., 2013; Farzana et al., 2016; Gromova et al., 2018). In zebrafish Mauthner neurons, Neurobeachin loss results in the failure of Connexin and electrical synapse scaffold ZO1 localization. Intriguingly, Neurobeachin is both necessary and sufficient postsynaptically for electrical synapse formation in this circuit (Miller et al., 2015). This supports a model wherein Neurobeachin controls the polarized trafficking of electrical components within the postsynaptic dendrite, although the molecular mechanism remains unknown. It is attractive to speculate that perhaps Neurobeachin acts to define dendritically targeted vesicles carrying electrical synapse cargo and that it may bridge them to the motor proteins required for postsynaptic delivery. Future experiments are required to identify how Neurobeachin functions in the dendrite to control synapse formation. The coordination of electrical and chemical synapses through a master synapse regulator such as Neurobeachin has critical implications for understanding the etiology of neurodevelopmental disorders (further discussed at the end of this review).

Once arriving at the synapse, Connexin vesicles must undergo exocytosis to become inserted into the membrane, allowing them to find their partner hemichannels in the neighboring neuron. Chemical synapses use v-SNAREs, present on pre- and postsynaptic vesicles, to bind t-SNAREs on the neuronal membrane and fuse the vesicles at the synapse. Work in goldfish Mauthner neurons examined the effect of SNAP-25 peptides, which block the formation of SNARE-complexes, on the mixed electrical-chemical synapses of the Mauthner club endings (Flores et al., 2012). Mixed electrical-chemical synapses at single synaptic termini represent another fascinating synaptic organization, and each component appears to be separately organized (Pereda, 2014; Nagy et al., 2019). Intra-dendritic application of these SNAP-25 peptides reduced both the electrical and the glutamatergic component of synaptic transmission suggesting the SNARE complex may function in Connexin insertion at the membrane (Flores et al., 2012). If the SNARE complex functions to fuse Connexin vesicles, there must be v-SNARE proteins within Connexin vesicles. But again, the composition of Connexin-containing vesicles and its protein constituents remain unknown. Insight into the molecular control of Connexin vesicle trafficking and membrane insertion in neurons will be critical to understanding electrical synapse formation and plasticity.

Further insights into the cell biological framework of electrical synapses will require an identification of the type of vesicles that contain Connexins; the motor, adaptor, and vesicle fusion proteins required for their transport and membrane fusion; and to determine if these features change between electrical synapse formation and plasticity. The elucidation of the cell biological pathways regulating electrical synapse protein trafficking will reveal whether they are the same or distinct from those of chemical synapses. The fact that electrical and chemical synapses have known distinct protein constituents suggests that at least some components will be unique, but the involvement of both Neurobeachin and SNAP-25 suggests some molecular overlap is also present. Besides, several trafficking conundrums remain. If Neurobeachin manages the postsynaptic trafficking of Connexins, what guides Connexin to the axon and the presynapse? And, in mammals, given that Cx36 is used within both the axon and the dendrite, how does a neuron resolve specific trafficking to these compartments? One possibility is that Connexin trafficking depends upon posttranslational modifications to the protein, such as phosphorylation (Li et al., 2009, 2013), to direct its localization. Or instead, Neurobeachin and other adaptor proteins may bind a scaffold protein which traffics with Connexin, as is observed with chemical synapse components (Tao-Cheng, 2007; Vukoja et al., 2018). Thus, cell-type-specific expression of these scaffolds and adaptors could result in different trafficking patterns and thus different cell biological construction of electrical synapses. This leads us to our next question: how do electrical synapse scaffolds control electrical synapse development?

## Organizing The Growing Electrical Synapse

To fully appreciate electrical synapse cell biology, we must understand that each electrical synapse is composed of plaques of tens to thousands of gap junction channels (Flores et al., 2012; Rash et al., 2012, 2013, 2015; Yao et al., 2014). These plaques of gap junction channels can take on many different conformations such as wide or thin ribbons and large circular regions of channels, either densely collected or with lace-like holes (Nagy et al., 2018). Connexins arrive at the synapse as hemichannels that are inserted at the boundaries of existing gap junction plaques where they then find a partner hemichannel in the adjoining neuron. Over time, the channels migrate towards the center of the plaque where they are endocytosed and sent to the lysosome for degradation (Lauf et al., 2002; Flores et al., 2012; Wang et al., 2015). The half-life of Cx36 is estimated to be between 1 and 3 h *in vivo*, so to maintain the electrical synapse, Cx36 must continuously be made and trafficked to the correct location (Flores et al., 2012; Wang et al., 2015). The known organizational principles of the plaque, and the turnover demand of Connexins, requires complex and ongoing molecular machinery to ensure appropriate development and homeostasis. But what ensures that the components of the electrical synapse, including Connexins, unite at the same place over time?

The gene *tjp1* encodes the ZO1 protein, a membrane-associated guanylate kinase (MAGUK) historically known for its necessity at tight junctions (Umeda et al., 2006) and epithelial gap junctions (Singh et al., 2005; Bao et al., 2019), and first identified at electrical synapses in the mouse brain (Li et al., 2004; Penes et al., 2005). Recent work in zebrafish shows that ZO1 is required for electrical synapse formation (Marsh et al., 2017) as larval fish mutant for the ZO1 homolog *tjp1b* lack Connexin localization resulting in functional deficits at electrical synapses. This suggests Tjp1b/ZO1 is required to either recruit, traffic, or stabilize Connexins at electrical synapses. Strikingly, the broad class of MAGUK scaffold proteins are well-known for their ability to aggregate protein components at other well-studied cell-cell junctions (see Figures 2B–E, MAGUKs shown in pink). For example, PSD95, SAP102, and PSD93 are all postsynaptic MAGUK proteins that localize at glutamatergic chemical synapses, make up a majority of proteins in the postsynaptic density, and interact either directly or indirectly with glutamatergic neurotransmitter receptors. Simultaneous knock-down of these three scaffolds results in smaller postsynaptic densities and a substantial reduction in chemical synapse transmission (Chen et al., 2015). These findings support MAGUKs, including ZO1, as master organizers of intercellular junctions. The unique features that facilitate their shared function at different cell-cell adhesions are exhaustively reviewed elsewhere (e.g., Zhu et al., 2016; Ye et al., 2018), but we will highlight several key characteristics that inform our understanding of ZO1 at the electrical synapse.

First, MAGUK proteins contain one or more PDZ (PSD95, Dlg1, and ZO1) domains. These domains interact with short ligand sequences, called PDZ binding motifs (PBMs), usually found at the C-terminus of the interacting protein. At cell-cell junctions, MAGUK PDZ domains bring together the C-termini of transmembrane (or auxiliary) proteins to create a carefully organized hub of molecular interactions (reviewed in Lee and Zheng, 2010). Although all PDZs share a canonical structure, amino acid differences in the binding surface of the PDZ and PBM confer interaction specificity (Giallourakis et al., 2006; Liu et al., 2019). Additionally, these specific interactions can be regulated by posttranslational modifications to either the PDZ or the ligand motif. At the electrical synapse, Cx36 and its teleost homologs all contain a C-terminal SAYV motif that interacts directly with the first PDZ domain of ZO1 (Li et al., 2004; Flores et al., 2008). It has, therefore, been proposed that electrical synapse formation and function requires a ZO1-PDZ1/Cx36-PBM interaction, but this has yet to be explicitly shown *in vivo*.

Second, in addition to transmembrane proteins, MAGUKs also interact with other scaffolds, regulatory proteins, signaling proteins, the cytoskeleton, and even in some cases the plasma membrane. This array of interactions allows MAGUKs to aggregate the pieces necessary to create, maintain, and regulate a functional junction. ZO1 is found in complex with numerous proteins found at the electrical synapse including neuronal Connexins (Li et al., 2004; Flores et al., 2008), CAMKII, which is responsible for some forms of electrical synapse plasticity (Alev et al., 2008; Flores et al., 2010; Li et al., 2012), and actin (Fanning et al., 2012). Thus, ZO1 appears poised to act as the central hub for electrical synapse protein organization and to act as a direct link to the cytoskeleton, yet the details of how it achieves this molecular coordination remain unknown.

Finally, recent studies have shown that many MAGUK proteins are capable of phase separating, creating dynamic and selective non-membrane bound organelles. All MAGUKs include a PDZ-SH3-GUK (PSG) tandem set of domains that function in regulated oligomerization (Pan et al., 2011; Rademacher et al., 2019), thus creating highly concentrated nanodomains that can aggregate various proteins to a specific site within a cell. At chemical synapses, phase separation within the presynaptic active zone clusters synaptic vesicle fusion proteins while at the postsynaptic density phase separation concentrates neurotransmitter receptors (reviewed in Chen et al., 2020). Recent work has found that ZO1 is capable of phase separation facilitated by its PSG tandem, and loss of ZO1’s phase separating capabilities in mammalian cell culture and the larval fish results in a loss of aggregation near the epithelial membrane and impairments in tight junction integrity (Beutel et al., 2019; Schwayer et al., 2019). Thus, it is attractive to propose a model of electrical synapse formation led by ZO1 phase separation which provides a local, specialized domain to capture Connexins and other molecular machinery through both direct and indirect interactions. This presents an exciting new avenue for future exploration.

Our knowledge of ZO1 and other MAGUKs at cell-cell junctions suggests a model in which ZO1 is oligomerized into nanodomains at the cell membrane destined to become Connexin plaques. As Connexins are rapidly turned over throughout the life of the electrical synapse, ZO1 stabilizes them, aggregates necessary regulatory proteins such as kinases, and links the structure to the cytoskeleton. Intriguingly, ZO1 has been shown to interact with numerous neuronally expressed Connexins, in addition to Cx36, suggesting that this mechanism may be common across all electrical synapses (reviewed in Hervé et al., 2012). The emerging evidence suggests ZO1 acts as a master organizer of electrical synapses once it is recruited to the site of the future electrical synapse. This, however, leads us to the question: what tells ZO1 where the electrical synapse should be?

## Specifying When and Where Electrical Synapses Are Created

Although it is possible that site specification initially occurs *via* extracellularly secreted signals, we know that synaptic initiation and maintenance requires cell adhesion molecules (CAMs). These membrane-spanning proteins have extracellular domains allowing for intercellular interactions with CAMs on an opposing cell. Additionally, they have intracellular domains that interact with the cytoskeleton, scaffolds, and other proteins that can trigger signaling cascades and the recruitment of other molecules. Thus, it is highly likely that neurons use CAMs to choose the right place and the right time to create an electrical synapse.

Could the Connexin proteins act as the CAM for electrical synaptogenesis? Connexins are indeed CAMs, and, in certain circumstances such as radial migration of neurons in the mouse cortex, the adhesive properties appear to be more important than the channel itself (Elias et al., 2007). So it is tempting to question if Connexins coordinate the recruitment of ZO1 and other required proteins to the electrical synapse. The gap junction channel as director of synapse formation appears to be the case in the leech, where the diversity of gap junction forming Innexin proteins drives the site-specific formation of electrical synapses (Baker and Macagno, 2014). However, in vertebrates, which use Connexins for their gap junctions, this may not be the case. In *Cx36* mutant mice that lack many neuronal gap junctions, electron microscopic analysis of the stereotyped dendro-dendritic electrical connections between olivary neurons found recognizable intercellular junctions still formed, but they lacked the classic electron-dense, gap junction morphology (De Zeeuw et al., 2003). A similar conclusion was found using immunohistochemistry at the MesV nucleus in *Cx36* null mice, where the stereotyped electrical synapse lacked neuronal Connexin staining, yet ZO1 was still localized to the putative electrical synaptic sites (Nagy and Lynn, 2018). Taken together, these results suggest that electrical synapses are specified by mechanisms other than Connexins, yet the nature of the signal remains unknown.

So what are the CAMs that specify electrical synapse sites? Vertebrate genomes contain thousands of genes that encode CAMs (Zhong et al., 2015), making it no small feat to identify the correct molecules that initiate electrical synapse site specification. Yet particular CAMs, such as the Nectins, may be the key as they play a critical role in establishing initial cell-cell adhesions and are known for their instructive role in adherens junction and tight junction formation in epithelia. At these locations, they precede the cadherin-based or claudin-based adhesions that are recruited later to these sites. Nectins build up a macromolecular complex by interacting with Afadin, an intracellular scaffold that directly interfaces with the actin cytoskeleton and other important scaffolds, such as alpha-catenin and ZO1, required for adherens junction and tight junction formation respectively (Yamada et al., 2006; Ooshio et al., 2010). In neurons, the loss of Nectins results in altered axon targeting whereas loss of Afadin results in greatly decreased neuronal N-cadherin and β- and αN-catenin puncta along with extensive reductions in excitatory synapse density (Honda et al., 2006; Beaudoin et al., 2012). The effects on electrical synapses have not been assessed. The relationship between Nectins and Afadins is likely cell type-specific, but these results support that, much like at tight junctions, these complexes form initial adhesions that lay a foundation for cadherin recruitment to the synaptic site.

But are Nectins responsible for specifying the locations of electrical synapses? Cx36, ZO1, and Afadin, but not Nectin, colocalize at electrical synapses in the rat/mouse brain. Moreover, Cx36 co-immunoprecipitates with Afadin in both whole-brain and retinal homogenates (Li et al., 2012), most likely through direct interaction with ZO1. Adjacent to electrical synapses, Afadin is also present at adherens junctions where it colocalizes with Nectin and N-cadherin (Li et al., 2012; Nagy and Lynn, 2018). This suggests a potential model where initial Nectin/Afadin adherens junctions form between neurons before electrical, or chemical, synapse formation and they recruit in cadherins to maintain the synapse, however, this has not been explicitly tested. How specification proceeds to differentiate between these future structures to guide specific molecular complex formation or whether these are causally required for formation remains unclear.

Alternatively, electrical synapses may use different complements of CAMs in their formation and maintenance, and to potentiate their functional plasticity. Chemical synapses use a multitude of synaptic CAMs not only to specify separate synaptic types (e.g., excitatory and inhibitory) but also to solidify and modulate synapse connections between neurons over time (Jang et al., 2017; Rawson et al., 2017). Other CAMs, such as claudins, occludins, and N-cadherin, all are found to interact with Connexins in epithelia alluding to their potential roles at the electrical synapse (reviewed in Hervé et al., 2012). However, attempting to elucidate the requirement of these CAMs *in vivo* is difficult due to the pleiotropic nature of these proteins and their use at many cellular junctions. So how can the electrical synapse CAMs be identified and studied? Zebrafish offer some advantages, particularly given the new methods in CRISPR-based reverse genetic screening (Shah et al., 2015), which provides a fast method for knocking out a large battery of potential CAMs to identify those that regulate electrical synapses. For the field, identifying the CAMs that specify the temporal and spatial electrical synapse dynamics is an essential hurdle that needs to be overcome to move forward in understanding the cell biology of the electrical synapse.

## Discussion and Conclusion

Here we have explored several critical open questions surrounding the cell biology of the electrical synapse. Filling these gaps in knowledge will greatly impact our understanding of the development and homeostasis of electrical synapses and will provide new frontiers in regards to the etiology of neurological disorders.

Numerous human disorders are characterized by the loss of gap junction channels, and they span tissues including the skin, heart, joints, teeth, and immune system, to name just a few (Jongsma and Wilders, 2000; van Steensel, 2004; Kleopa and Scherer, 2006; Laird, 2006, 2010; Wong et al., 2017; Donahue et al., 2018). Indeed, the leading cause of deafness is due to the loss of Connexins expressed in the ear, which is currently, and extremely controversially, earmarked for a possible human CRISPR trial (Batissoco et al., 2018; Cyranoski, 2019). These pathologies seemingly emerge from the disruption of wide-ranging gap junction roles within cell proliferation and differentiation, morphogenesis, cell migration, growth control, and many other cell biological processes (McGonnell et al., 2001; Vinken et al., 2006; Kardami et al., 2007; Marins et al., 2009). If we turn our gaze to the nervous system, we find that in *Cx36* knockout mice there are brain-wide electrical synapse defects such as within the cerebellum where motor function is impaired, in the hippocampus where perturbed long-term potentiation and network oscillations impact learning and memory, in the cortex where cortical interneurons become desynchronized, and in both visual and olfactory systems which are dysfunctional (Güldenagel et al., 2001; Frisch et al., 2005; Bissiere et al., 2011; Wang and Belousov, 2011; Zolnik and Connors, 2016; Pouille et al., 2017). Similar disruptions are mirrored in zebrafish, where elimination of *Cx36* homologs results in delayed responses to threatening stimuli and motor coordination defects (Miller et al., 2017). These behavioral defects in animal models lacking a broad class of electrical synapses are exactly what the field of neurodevelopment would expect for genes linked to disease phenotypes (Mas et al., 2004; Hempelmann et al., 2006; Solouki et al., 2010; Li et al., 2015; Kunceviciene et al., 2018). Namely, that many disorders of neurodevelopment result not in large effects with gross dysfunction, but instead are comprised of subtle molecular differences that slightly shift the functional outcomes. Indeed, many so-called synaptopathies are thought to affect synapse formation and perturb excitatory/inhibitory balances (Grant, 2012). We suggest that the perspective should be broadened to the electrical/excitatory/inhibitory balance, as disruptions to any of these components lead to subsequent abnormal circuit function which develops to have larger behavioral ramifications over time. Indeed, electrical synapse disruptions are proposed to contribute to the etiology of disorders such as autism (Welsh et al., 2005) and epilepsy (Cunningham et al., 2012). However, Connexin loss is not yet a well-appreciated contributor to such disorders. We think it is likely that the growing awareness and attention electrical synapses are receiving in neural circuit formation, function, and behavior will bring to light their links to a large set of neurodevelopmental disorders.

In this review, we have made the case that Connexins are not the full story in considering the form and function of the electrical synapse. Indeed, our work on Neurobeachin, which itself is linked with both autism and epilepsy in human patients, suggests that as we begin to understand the totality of electrical synapse formation, how these structures are related to disorders of neural function will become ever more apparent. Therefore, we fundamentally need to expand our understanding of the cell biological mechanisms that develop, maintain, and regulate electrical synapses. And we need to improve our knowledge of the mechanistic relationship between electrical and chemical synapse formation to clarify the contributions of each synapse type to development and adult neural circuit function. In conclusion, we predict that the continuing studies of electrical synapse structure and function will provide a new framework for understanding fundamental mechanisms of brain structure and function as well as the etiology of the disease.

## Author Contributions

EM, AL, and AM discussed and wrote the review. All authors contributed to manuscript revision, read and approved the submitted version.

## Conflict of Interest

The authors declare that the research was conducted in the absence of any commercial or financial relationships that could be construed as a potential conflict of interest.
